# Preoxygenation in the ICU

**DOI:** 10.3390/jcm14207305

**Published:** 2025-10-16

**Authors:** Clément Monet, Mathieu Capdevila, Inès Lakbar, Yassir Aarab, Joris Pensier, Audrey De Jong, Samir Jaber

**Affiliations:** 1 Department of Anaesthesiology and Critical Care Medicine, Saint-Eloi Hospital, University Teaching Hospital of Montpellier, 80 Avenue Augustin Fliche, 34295 Montpellier, France; 2 PhyMedExp, INSERM U1046 Montpellier, Université de Montpellier, 34295 Montpellier, France

**Keywords:** tracheal intubation, intensive care, preoxygenation, hypoxemia, non invasive ventilation, high flow nasal cannula

## Abstract

Tracheal intubation is a frequent and high-risk procedure in the intensive care unit (ICU). Unlike elective intubation in the operating room, ICU intubation is often performed under emergent conditions in physiologically unstable patients, leading to increased technical difficulty and higher complication rates. Among these, hypoxemia is particularly frequent and represents a major determinant of morbidity and mortality. Optimizing preoxygenation is therefore a cornerstone of safe airway management in critically ill patients. The aim of this review is to explore the advantages and limitations of each preoxygenation strategy and to provide clinicians with clear, practical guidance to optimize airway management in the ICU. Preoxygenation aims to increase oxygen reserves in order to prolong the duration of safe apnea. Conventional methods include high-flow oxygen delivery through a tightly fitted face mask, though efficacy depends on minimizing leaks. More advanced strategies include non-invasive ventilation (NIV), which improves both alveolar oxygen fraction and lung volume, and high-flow nasal cannula (HFNC), which additionally allows apneic oxygenation during intubation. Randomized controlled trials, including the recent PREOXY study, demonstrate the superiority of NIV over facemask preoxygenation in reducing peri-intubation desaturation, particularly in hypoxemic patients. HFNC is valuable when NIV is contraindicated, while combined approaches (NIV plus HFNC) may further enhance efficacy. Beyond technique, structured protocols and team organization are crucial to reduce complications. In conclusion, preoxygenation is an essential, patient-specific intervention that mitigates the risks of ICU intubation. Familiarity with available methods enables clinicians to tailor strategies, optimize oxygenation, and improve patient safety during this high-risk procedure.

## 1. Introduction

Tracheal intubation remains a cornerstone of critical care management in the ICU. Mechanical ventilation—requiring tracheal intubation—represents the most frequently initiated form of organ support in critically ill patients [1]. Despite considerable progress in airway management over the last decades, tracheal intubation in the ICU continues to pose significant challenges for clinicians and carries a substantial risk of life-threatening complications. These include severe hypoxemia, cardiovascular collapse, cardiac arrest, esophageal intubation, arrhythmias, agitation, and aspiration pneumonia—none of which are rare in this setting [2,3,4,5]. Among these complications, hypoxemia is one of the most frequent and feared complication observed in critically ill adults undergoing tracheal intubation [3,6]. The high-risk nature of ICU intubation stems from a combination of factors, including unstable clinical conditions, profound physiological modifications, and the urgency with which the procedure is usually performed [7]. Unlike elective intubation in the controlled environment of the operating room (OR), ICU intubation is typically carried out under emergent circumstances, often in patients with some combination of respiratory, circulatory, or neurologic failure.

In an observational study of 208 patients who underwent both OR and ICU intubation within the same month, ICU intubations were associated with poorer glottic visualization, lower first-pass success rates, higher complication rates, and more frequent difficult airway management compared with elective intubation [8]. Consistently, other studies have confirmed that airway management in the ICU is associated with greater technical difficulty and higher complication rates than in the OR [9,10].

Several pathophysiological and clinical factors contribute to these challenges. Critical illness and common ICU interventions may negatively impact airway anatomy and physiology. For instance, aggressive fluid resuscitation, capillary leak syndrome, prone positioning, or a history of prolonged intubation can all lead to upper airway edema or structural distortion, complicating both laryngoscopy and intubation attempts [11,12,13,14,15]. Large-scale international epidemiological studies have provided valuable insights into the contexts in which ICU intubations are performed, as well as the nature and frequency of associated complications [3,9,16]. These data consistently show that severe complications occur in approximately 38–45% of ICU intubations. The incidence of difficult intubation varies widely, ranging from 8% to 20% depending on the population studied and methodology [4,5,17,18]. This variability reflects differences in definitions, clinical settings, operator experience, and patient characteristics. In a large prospective multicenter study of 1400 ICU intubations, the incidence of difficult intubation was 10.35%. Importantly, in that cohort, difficult intubation was associated with a 51% rate of life-threatening complications, compared to 36% in the overall ICU population [19]. Beyond mechanical difficulty, the concept of “physiologically difficult airway”—characterized by severe hypoxemia, shock, metabolic acidosis, or right ventricular failure—represents an additional layer of complexity that requires anticipation and tailored management strategies [7].

Optimizing pre-intubation management is therefore critical. De Jong et al. identified several independent risk factors for peri-intubation cardiac arrest, including arterial hypotension, hypoxemia, obesity, age over 75 years, and absence of preoxygenation [2], underscoring the importance of careful preparation before airway instrumentation.

In addition to patient-related risks, procedural and organizational factors also strongly influence outcomes. Operator experience, the immediate availability of airway adjuncts (e.g., videolaryngoscopes, supraglottic devices, or bougies), and the presence of a trained multidisciplinary team all play essential roles [20]. Recent studies have emphasized the benefit of structured approaches, including pre-intubation checklists [21], predefined rescue strategies, and team briefings, which together improve first-pass success and mitigate complications [5]. Simulation-based training further enhances both technical and non-technical skills, preparing clinicians to handle high-stakes scenarios under stress. Technological innovations such as videolaryngoscopy have improved glottic visualization and may increase first-pass success compared to direct laryngoscopy [22].

Importantly, attention must extend beyond the mechanics of laryngoscopy to the physiological preparation of the patient—particularly oxygenation. Peri-intubation hypoxemia is often the immediate cause of major adverse events and is closely linked to poor outcomes. Preoxygenation is therefore a cornerstone of safe airway management, aiming to maximize oxygen reserves and prolong the duration of “safe apnea.” Recent advances such as high-flow nasal oxygen (HFNC), non-invasive ventilation (NIV), and apneic oxygenation have expanded the armamentarium available to intensivists, offering physiological advantages that may significantly reduce hypoxemia during intubation. Structured bundles integrating pre-, peri-, and post-intubation interventions—including systematic preoxygenation—have demonstrated improved patient outcomes and are increasingly recommended by expert consensus statements [23,24,25].

Despite its fundamental role, significant knowledge gaps remain regarding the optimal approach to preoxygenation and peri-intubation oxygenation in the ICU. Existing studies are heterogeneous in terms of patient populations, techniques, and endpoints, and many focus on surrogate outcomes (e.g., lowest SpO_2_) rather than patient-centered outcomes such as mortality, neurological injury, or length of stay. Moreover, the relative efficacy, feasibility, and safety of emerging strategies—alone or in combination—remain incompletely defined. Finally, there is limited evidence on how preoxygenation should be individualized based on underlying physiology, or how it should be integrated into structured airway management protocols.

This review aims to address these gaps by synthesizing current evidence on preoxygenation and peri-intubation oxygenation strategies for tracheal intubation in critically ill patients. We discuss the physiological principles underlying preoxygenation, summarize the evidence supporting conventional and novel approaches, and highlight practical considerations for their implementation in the ICU. In doing so, we also aim to identify areas where further research is needed and provide a framework for optimizing oxygenation and improving patient safety during airway management.

## 2. Rationale for Preoxygenation

Preoxygenation is a crucial step to prevent per-intubation hypoxemia in the ICU. Its primary goal is to maximize oxygen stores and thereby delay the onset of arterial oxyhemoglobin desaturation during apnea—commonly referred to as increasing the duration of “safe apnea”. While desirable in all patients, preoxygenation is indispensable in critically ill individuals in whom the “cannot intubate, cannot ventilate” scenario is difficult to predict and carries a high risk of severe complications. Moreover, as highlighted in the introduction, ICU patients face an intrinsically higher risk of life-threatening complications during airway management, particularly hypoxemia.

From a physiological standpoint, preoxygenation consists of delivering pure oxygen to the patient in order to replace nitrogen within the lungs and increase their oxygen reserves [26,27]. These reserves are mainly located in the lungs, and to a much lesser extent in the blood and tissues. They depend on the functional residual capacity (FRC)—the volume of gas remaining in the lungs after a normal exhalation—and the alveolar fraction of oxygen (FAO_2_), defined as the concentration of oxygen in the alveoli [28]. In healthy individuals, breathing pure oxygen (inspired oxygen fraction of 1) increases the FAO_2_ to approximately 90%, while the fraction of alveolar nitrogen (FAN_2_) decreases accordingly. Thus, for an FRC of 2500 mL, the stored oxygen volume exceeds 2000 mL [29].

When evaluating preoxygenation, both efficacy and efficiency must be considered. Efficacy refers to the physiological ability of a technique to achieve alveolar denitrogenation and maximize oxygen stores, as influenced by factors such as FiO_2_, interface seal, preoxygenation duration, and the application of positive airway pressure. Efficiency, in contrast, reflects the interaction between the oxygen reservoir and the patient’s systemic oxygen demand, determined by FRC, arterial oxygen content, cardiac output, and whole-body oxygen consumption (VO_2_). In practice, efficacy determines the potential to increase oxygen reserves, while efficiency dictates how long these reserves can sustain adequate oxygenation during apnea. Thus, techniques must be assessed not only for their ability to enrich alveolar oxygen but also for their capacity to maintain a favorable balance between oxygen supply and consumption (Table 1). Monitoring the effectiveness of preoxygenation can be achieved using the expired oxygen fraction (FeO_2_) or the end-tidal oxygen concentration (EtO_2_), although these measurements are rarely available in the ICU. An FeO_2_ greater than 90% reflects adequate denitrogenation and a high FAO_2_. However, it does not account for the FRC and therefore does not guarantee the absence of desaturation during the procedure. The higher the patient’s risk of peri-procedural hypoxemia, the more critical it becomes to optimize preoxygenation.

All ICU patients requiring intubation are at risk, but several groups deserve special attention. Obesity, pregnancy, acute or chronic respiratory diseases, and predicted difficult intubation are well recognized risk factors. In obese patients, FRC decreases as body mass index rises, leading to markedly shorter apnea time without desaturation [30]. Strategies to counteract this loss in lung volume—such as head-up positioning, positive airway pressure, or recruitment maneuvers—are often necessary to achieve adequate preoxygenation [30]. Similarly, in pregnancy, decreased FRC and increased oxygen consumption combine to shorten safe apnea time, making preoxygenation particularly challenging. Beyond these anatomical and physiological factors, certain comorbidities reduce tolerance to even moderate hypoxemia, including coronary artery disease, epilepsy, and sickle cell disease. In such patients, the threshold for clinically significant complications may be reached within seconds of desaturation, underscoring the importance of maximizing oxygen reserves before airway instrumentation.

**Table 1:** Factors linked to efficacy and efficiency of preoxygenation. Adapted from Baraka AS, Salem MR. Preoxygenation. In: Benumof and Hagberg’s Airway Management, 3rd ed., edited by Hagberg CA (Elsevier, 2013), with permission [31].

**Efficacy**
Inspired oxygen concentration
Presence of leak
Patient-ventilation interface
Fresh gas flow
Tidal volume vs. deep breathing
Duration of preoxygenation
Alveolar ventilation/functional residual capacity ratio
Positive pressure (NIV or CPAP vs. Facemask or HFNC)
**Efficiency**
Oxygen volume in lungs
FRC
Alveolar oxygen tension
System oxygen supply vs. demand balance
Arterial oxygen content
Cardiac output
VO_2_ (whole body oxygen consumption)

NIV: non-invasive ventilation, CPAP: continuous positive airway pressure, HFNC: high-flow nasal cannula, FRC: functional residual capacity, VO_2_: whole body oxygen consumption.

Finally, the ICU environment introduces additional challenges compared with the operating room. Many patients are uncooperative, agitated, or already severely hypoxemic, limiting the effectiveness of standard face mask preoxygenation. Mask leaks, high inspiratory demand, or shunt physiology due to lung injury can all compromise oxygen uptake despite high inspired oxygen fractions. In these situations, advanced techniques such as non-invasive ventilation or high-flow nasal oxygen may offer physiological advantages by combining denitrogenation with alveolar recruitment and improved oxygen delivery. These modalities, as well as the concept of apneic oxygenation, have therefore attracted growing interest as complementary or alternative strategies for preoxygenation in the ICU.

## 3. Strategies for Pre-Oxygenation

**Figure 1:**
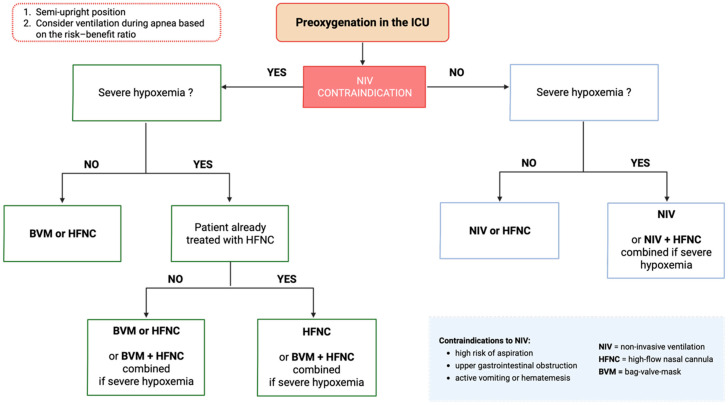
Preoxygenation algorithm. ICU: Intensive care unit, NIV: non-invasive ventilation, HFNC: high-flow nasal cannula, BVM: bag–valve–mask.

Several strategies are available for preoxygenation of critically ill patients (Figure 1 and Table 2). These include conventional facemask methods (non-rebreather mask or bag–valve–mask, [BVM]), high-flow nasal cannula (HFNC), continuous positive airway pressure (CPAP), non-invasive ventilation (NIV) with inspiratory pressure support and PEEP, and combined modalities such as NIV plus HFNC or facemask plus HFNC.

Each approach has distinct physiological effects, technical requirements, and limitations. Regardless of the method, patients should be positioned semi-upright (20–30°) rather than supine, as this improves preoxygenation efficiency by increasing functional residual capacity (FRC). This positional adjustment is especially critical in obese patients, where FRC is markedly reduced in the supine position.

### 3.1. Conventional Preoxygenation

“Conventional” or standard preoxygenation involves 3 to 5 min of spontaneous breathing with FiO_2_ 1.0 delivered through a tightly applied face mask. The effectiveness of this technique depends heavily on achieving an airtight seal; any leak allows entrainment of ambient air, thereby lowering inspired oxygen concentration. High oxygen flow rates (≥15 L/min) are recommended to reduce the impact of leaks and maintain FiO_2_ close to 1.0. Non-rebreather masks, although commonly used, are less effective than a well-applied BVM in achieving high expired oxygen fractions (FeO_2_) [20]. Differences in performance may also exist between BVM devices, further emphasizing the importance of equipment choice and operator familiarity [32]. While this technique is simple and widely available, its efficacy is limited in patients with severe hypoxemia or reduced FRC, making it insufficient in many ICU scenarios. A recent network meta-analysis confirmed that facemask preoxygenation is inferior to both NIV and HFNC, with a higher risk of hypoxemia during intubation [33].

### 3.2. Preoxygenation with Non-Invasive Ventilation (NIV)

NIV is a highly effective preoxygenation strategy that delivers two levels of pressure, thereby preventing derecruitment and atelectasis while increasing FRC. NIV thus simultaneously improves alveolar oxygen fraction (FAO_2_) and FRC, the two key determinants of oxygen reserve. As early as 2006, a randomized controlled trial demonstrated the superiority of NIV over standard face mask preoxygenation in hypoxemic ICU patients [34]. More recently, the large PREOXY trial, published in 2024 in the New England Journal of Medicine [35], confirmed this benefit in a broad, unselected ICU population. In that study, desaturation below 85% SpO_2_ between induction and 2 min after intubation occurred in 9.1% of patients in the NIV group versus 18.5% in the face mask group, without an increased incidence of aspiration events. In this study, the occurrence of vomiting, hematemesis, hemoptysis, or epistaxis were among exclusion criteria. Therefore, the conclusions of this study cannot be applied to these situations. In such patients, NIV remains contraindicated.

When comparing NIV and HFNC, data suggest similar efficacy in patients with mild hypoxemia, but NIV seems to provide superior protection against desaturation in moderate-to-severe hypoxemia (PaO_2_/FiO_2_ < 200) [36]. The physiological rationale lies in the ability of NIV to generate PEEP and recruitment, which HFNC alone cannot consistently achieve.

To maximize alveolar recruitment while avoiding barotrauma or hemodynamic compromise, careful titration of NIV settings is essential. In most clinical situations, pressure support levels between 5 and 10 cmH_2_O combined with a positive end-expiratory pressure (PEEP) of approximately 5 cmH_2_O are recommended. The objective should be to achieve expired tidal volumes around 6–8 mL/kg of predicted body weight [28,37]. Adjustments should be individualized based on oxygenation targets, patient tolerance, and the degree of respiratory effort.

Limitations of NIV preoxygenation include patient tolerance, the need for tight-fitting masks, and the risk of gastric insufflation if high pressures are used. While NIV improves gas exchange, the application of positive pressure may also influence cardiovascular dynamics. Increased intrathoracic pressure can reduce venous return and subsequently decrease cardiac output, particularly in patients with relative hypovolemia or vasoplegia. Therefore, optimization of volume status prior to preoxygenation is strongly recommended, and vasopressor support should be anticipated in hemodynamically unstable patients. Continuous monitoring of blood pressure and heart rate during NIV preoxygenation is essential to ensure safety and to promptly address potential hemodynamic compromise [5,28].

Overall, NIV remains the most efficacious preoxygenation technique for critically ill patients, especially those with acute hypoxemic respiratory failure. Proper titration of pressures and careful hemodynamic monitoring are crucial to maximize its benefits and minimize potential risks.

### 3.3. Preoxygenation with High-Flow Nasal Cannula (HFNC)

HFNC is an alternative for patients in whom NIV is contraindicated—for example, in cases of vomiting, hematemesis, hemoptysis, or epistaxis [38]. A key advantage of HFNC is that it allows apneic oxygenation during the intubation procedure (see “per-intubation oxygenation”). In non-severely hypoxemic patients, HFNC has been shown as relatively superior to face mask preoxygenation [39] and non-inferior to NIV. However, in moderate-to-severe hypoxemic patients (PaO_2_/FIO_2_ < 200), NIV was associated with less occurrence of severe hypoxemia compared to HFNC [37]. Discrepancies between studies may be due to two factors: (1) heterogeneity in oxygen flow rates (ranging from 15 to 60 L/min), and (2) variable use of apneic oxygenation during intubation. Some expert guidelines recommend continuing HFNC during preoxygenation for patients already receiving this therapy, provided they are not severely hypoxemic. Overall, HFNC remains a valuable option for patients who are non-hypoxemic or have contraindications to NIV.

### 3.4. Combined Preoxygenation Strategies

An innovative strategy combining NIV and HFNC simultaneously, leveraging the benefits of both positive pressure and high oxygen flow, has also been described as an alternative method of preoxygenation [40]. In a randomized trial involving 49 hypoxemic ICU patients, this strategy was shown to be superior to NIV alone in preventing desaturation during intubation. A key takeaway is that these techniques should not be viewed as mutually exclusive, but rather combined and tailored based on patient-specific characteristics, underlying pathology, and team expertise. In the same line, a preoxygenation strategy combining high-flow nasal oxygen and a face mask has also been evaluated in the operating theatre setting. This strategy, which still requires evaluation, could also provide benefits in the intensive care setting [41].

### 3.5. Sedation-Assisted Preoxygenation

A particular challenge in the ICU is the agitated or uncooperative patient, in whom standard preoxygenation often fails due to mask leaks or poor tolerance. Sedation-assisted preoxygenation has recently emerged as a solution. In a randomized controlled trial, administration of a dissociative dose of ketamine (0.5–1.5 mg/kg) to facilitate tolerance of NIV or face mask preoxygenation significantly reduced the incidence of peri-intubation hypoxemia compared with standard rapid sequence induction alone [42]. This strategy is promising, but it requires expertise in ketamine use and careful patient selection to avoid delaying intubation in cases of imminent respiratory collapse.

**Table 2:** Suggested preoxygenation strategies by patient population and related key evidence.

Population	Strategy	Practical Settings	Key Evidence	Alternatives/Add-ons	Caveats
**Severe hypoxemia** (PaO_2_/FiO_2_ < 200)	NIV	PS ~5–10 cmH_2_O; PEEP ~5 cmH_2_O; FiO_2_ 1.0; tight mask	[33,34,35,37]	NIV + HFNC [43] If contra indications to NIV: HFNC or HFNC + BVM [41] Gentle mask ventilation during apnea [44] Apneic oxygenation [43]	Interface intolerance
**Moderate hypoxemia** (PaO_2_/FiO_2_ 200–300)	NIV or HFNC	HFNC 40–60 L·min^−1^, FiO_2_ 1.0; or NIV as above	[33,39,45]	Gentle mask ventilation during apnea [44] Apneic oxygenation [43]	Interface intolerance
**Mild or no hypoxemia**	NIV or HFNC or BVM	Facemask, reservoir; or HFNC as above or NIV as above	∅	∅	Leakage reduces FiO_2_
**Obesity** (BMI ≥ 30 kg·m^−2^)	NIV (Or HFNC)	PS 5–10 cmH_2_O + PEEP ~5 cmH_2_O; FiO_2_ 1.0; ramped position HFNC as above	[30,46,47] Warning: OR data extrapolated	NIV + HFNC or BVM + HFNC [41] if severe hypoxemia	∅
**High aspiration risk active vomiting**	HFNC	HFNC as above	Pragmatic safety choice	Apneic oxygenation [43]	Rapid sequence essential Avoid aggressive BVM
**Anticipated difficult** **laryngoscopy**	NIV +/− HFNC	NIV as above; HFNC as above	[33,34,35,37] Pragmatic safety choice	Gentle mask ventilation during apnea [44] Apneic oxygenation [43]	Ensure rescue plan

NIV: non-invasive ventilation, HFNC: high-flow nasal cannula, BVM: bag–valve–mask, PEEP: positive end-expiratory pressure; PS: pressure support; OR: operating room. ∅: not applicable.

## 4. Peri-Intubation Oxygenation

Peri-intubation oxygenation refers to techniques aimed at maintaining oxygenation during the interval between induction and the establishment of effective mechanical ventilation, including apneic oxygenation and ventilation delivered throughout laryngoscopy and tracheal intubation.

### 4.1. Apneic Oxygenation

Once the face mask or the NIV mask is removed to perform tracheal intubation (oral or nasal), clinicians lose an important advantage: continuous oxygen delivery. This interval—extending from mask removal until the initiation of positive pressure ventilation—represents a period of vulnerability, during which desaturation can develop rapidly. Apneic oxygenation aims to bridge this gap and prolong the duration of safe apnea, provided that certain physiological conditions are met: a patent airway down to the alveoli, a high alveolar oxygen pressure, and, crucially, prior denitrogenation of the lungs [48]. The latter step ensures that the functional residual capacity (FRC) is filled predominantly with oxygen rather than nitrogen, thereby maximizing oxygen reserves. The physiological principle underlying apneic oxygenation lies in the imbalance between alveolar oxygen uptake and carbon dioxide excretion. Because oxygen uptake exceeds CO_2_ elimination, a slight negative pressure develops within the alveoli. This gradient drives passive oxygen flow from the upper airways into the alveolar space, allowing oxygenation to continue even in the absence of active ventilation [49]. High-flow nasal oxygen (HFNC) is particularly well suited for this purpose, as it provides high inspired oxygen fractions, flushes nasopharyngeal dead space, and can be maintained throughout laryngoscopy without interfering with the procedure. Nevertheless, the efficacy of apneic oxygenation is highly dependent on baseline physiology. Patients with severe hypoxemia, large intrapulmonary shunt fractions, obesity, or profound lung injury may derive limited benefit, and desaturation may still occur within seconds despite optimal conditions. Current clinical studies suggest that while apneic oxygenation improves oxygenation in some groups, it does not eliminate the risk of desaturation in severely hypoxemic ICU patients [43].

### 4.2. Mask Ventilation During the Apneic Period

A complementary approach is gentle mask ventilation during the apneic period. Recent data indicate that carefully controlled ventilation between induction and laryngoscopy can reduce the incidence of peri-intubation hypoxemia without increasing aspiration risk [23]. In patients who are severely hypoxemic and without clear evidence of a full stomach, weighing the risk–benefit ratio between desaturation and aspiration often supports the use of this strategy. Notably, when preoxygenation has been performed with NIV, it is relatively straightforward to continue ventilation during the apneic phase if needed.

## 5. Intubation Protocol

**Figure 2:**
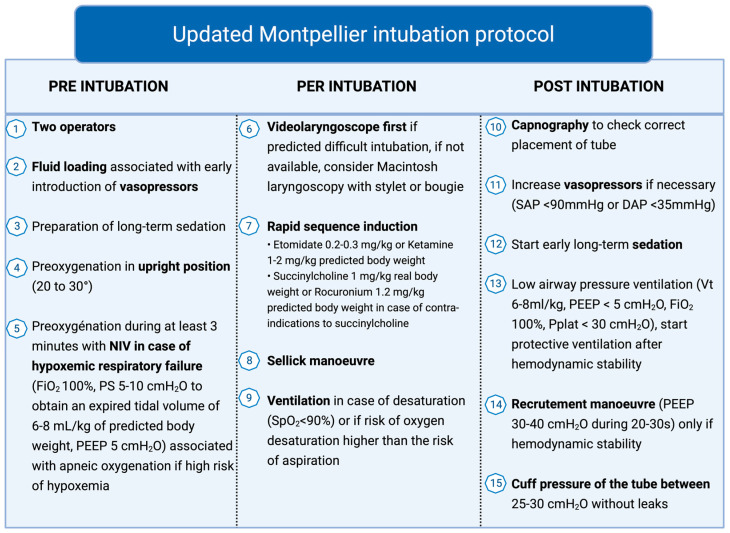
Updated Montpellier intubation protocol. The protocol integrates pre-, peri-, and post-intubation care. After [24,52]. PS: pressure support, PEEP: positive end-expiratory pressure, SAP: systolic arterial pressure, DAP: diastolic arterial pressure.

Beyond the choice of preoxygenation technique, a structured intubation protocol is essential in the ICU to reduce intubation-related complications. Such protocols provide a systematic framework that standardizes preparation, promotes teamwork, and reduces variability between operators. In addition to team organization and equipment selection, several other key elements must be anticipated: choice and dosing of anesthetic agents, the preoxygenation method to be applied, hemodynamic optimization with fluids and vasopressors, and the definition of a backup plan in case of failed airway management [24]. Over the last decade, several groups have advocated for the implementation of structured protocols and intubation bundles to standardize practice and reduce adverse events. Jaber et al. (2010) demonstrated in a prospective multicenter study that the introduction of a 10-item intubation bundle, including systematic preoxygenation using the most effective method available (e.g., NIV with PEEP in hypoxemic patients, combined with apneic oxygenation when appropriate), fluid loading, and team role allocation, significantly reduced the incidence of severe hypoxemia and life-threatening complications [5]. Similarly, Corl et al. (2018) reported improved team performance and adherence to safety steps after implementation of pre-intubation checklists [50]. Figure 2 illustrates the updated Montpellier protocol, highlighting preoxygenation as a mandatory step prior to induction and intubation.

Continuous monitoring and anticipation of post-intubation ventilation settings are also critical steps. Checklists, read aloud by the team leader just before the procedure, allow for final verification of essential equipment and drugs while ensuring that each critical step is executed in the correct sequence. Structured team communication and simulation-based training should complete these intubation protocols in order to maximize the probabilities of success and patient security. Nevertheless, the global evidence remains debated. A recent systematic review and meta-analysis of 11 studies did not find a consistent association between checklist use and improved outcomes such as first-pass success or mortality [51]. Importantly, this analysis reported heterogeneity across studies and suggested that methodological limitations may have underestimated the true benefit of structured protocols.

Beyond their measurable effect on hard clinical outcomes, intubation protocols and checklists provide undeniable advantages: they enhance team coordination, reduce cognitive load in stressful situations, and ensure systematic equipment readiness. These organizational and safety culture benefits justify their widespread adoption in daily ICU practice. In this context, protocols should not be regarded as optional tools but as essential components of patient safety strategies, complementing technical expertise and individualized clinical judgment. 

## 6. Conclusions

Preoxygenation and peri-intubation oxygenation strategies are central to improving the safety of airway management in critically ill patients. Current evidence clearly demonstrates that optimizing oxygen stores, using effective techniques such as non-invasive ventilation with PEEP or high-flow nasal oxygen, and integrating apneic oxygenation or gentle mask ventilation during the apneic phase significantly reduce the risk of severe desaturation. Structured airway management bundles, which embed pre-, peri-, and post-intubation interventions, further enhance patient safety and are increasingly endorsed in expert recommendations.

From a practical perspective, clinicians should individualize their approach based on patient physiology, severity of hypoxemia, and logistical constraints. In high-risk patients, strategies prioritizing efficacy—such as NIV preoxygenation and positive pressure—should be favored, whereas in unstable or uncooperative patients, efficiency and feasibility may be more important. Monitoring tools like end-tidal oxygen concentration (EtO_2_) and careful titration of ventilatory pressures can refine preoxygenation quality and mitigate complications.

Despite substantial progress, several key questions remain unanswered. The optimal combination of preoxygenation techniques (e.g., simultaneous NIV and HFNC, simultaneous bag–valve–mask and HFNC) and strategies to individualize preoxygenation based on lung mechanics or oxygen consumption require further investigation. Large, multicenter randomized trials are also needed to clarify the impact of standardized intubation bundles on patient-centered outcomes beyond hypoxemia, including mortality and neurological sequelae. Finally, addressing implementation barriers—such as training, equipment availability, and adherence to checklists—is crucial to translating evidence into routine practice.

In summary, preoxygenation is no longer a simple preparatory step: it is a critical safety intervention. Continued research, innovation, and standardization of practice will be essential to further reduce the risks associated with intubation in the intensive care unit.

## Data Availability

No new data were created or analyzed in this study. Data sharing is not applicable to this article.

## Abbreviations

FRC	Functional Residual Capacity
FAO_2_	Alveolar Fraction of Oxygen
HFNC	High-Flow Nasal Cannula
NIV	Non-Invasive Ventilation
PEEP	Positive End-Expiratory Pressure
FiO_2_	Fraction of Inspired Oxygen
SpO_2_	Peripheral Capillary Oxygen Saturation
PaO_2_	Arterial Partial Pressure of Oxygen
BMV	Bag–Valve–Mask
